# Some More Don’ts

**Published:** 1899-02

**Authors:** 


### Some More Don'ts.

The Don'ts extant at this time are numerous, but there is room for a few more of a different kind:

Don't fail to renew your subscription when the time has expired.

Don't fail to notify the publisher when you have changed your address.

Don't fail to notify the office when you discontinue.

Don't leave this duty to the postmaster.

Don't forget or neglect to do the gentlemanly thing.

Don't throw the statement of your account in the waste basket and leave the publisher under the impression that the statement was not received.

Don't fail to make note of it when a bill is presented for payment.

Don't conclude that no personal honor is involved in unpaid dues on subscription.

Don't discriminate between the debt due for your selected journal from one due you from your patient.

Don't forget that the golden rule is binding here as elsewhere.

And don't forget that money is required to conduct a medical journal.—*Medical Herald.*

